# First report of *Microtriatoma borbai* Lent & Wygodzinsky, 1979 (Hemiptera, Reduviidae, Triatominae) in the state of Espírito Santo, Brazil: would *M. borbai* be living in eucalyptus crops?

**DOI:** 10.1590/0037-8682-0147-2021

**Published:** 2021-06-02

**Authors:** Hélcio Reinaldo Gil-Santana, David dos Santos Martins, João Bosco da Silva, Jader de Oliveira

**Affiliations:** 1 Instituto Oswaldo Cruz, Laboratório de Diptera, Rio de Janeiro, RJ, Brasil.; 2 Instituto Capixaba de Pesquisa, Assistência Técnica e Extensão Rural, Vitória, ES, Brasil.; 3 Suzano S.A., Unidade Aracruz, Aracruz, ES, Brasil.; 4 Universidade de São Paulo, Faculdade de Saúde Pública, Laboratório de Entomologia em Saúde Pública, São Paulo, SP, Brasil.; 5 Universidade Estadual Paulista “Julio de Mesquita Filho”, Faculdade de Ciências Farmacêuticas, Laboratório de Parasitologia, Araraquara, SP, Brasil.

**Keywords:** Biogeography, Bolboderini, Kissing bugs

## Abstract

**INTRODUCTION:**

The occurrence of *Microtriatoma borbai* in the state of Espírito Santo, Brazil is reported by the first time.

**METHODS:**

A triatomine specimen collected in a hybrid eucalyptus crop in the municipality of Aracruz, Espírito Santo state was found to be a male *M. borbai*.

**RESULTS:**

This finding expands the geographical distribution of *M. borbai* from four to five Brazilian states. It is the first report of *M. borbai* occurrence inside a eucalyptus crop.

**CONCLUSIONS:**

The occurrence of *M. borbai* in the state of Espírito Santo broadens the geographical distribution of this species in southeastern Brazil.

All species included in the subfamily Triatominae are blood-sucking bugs; they are either proven or potential vectors of Chagas disease caused by the protozoan *Trypanosoma cruzi* (Chagas, 1909) (Kinetoplastida, Trypanosomatidae). Chagas disease is a major public health issue in Latin America^1^ ^,^ ^2^. 

Currently, the subfamily Triatominae includes 156 species, grouped into five tribes^3^ ^,^ ^4^ ^,^ ^5^ ^,^ ^6^. The tribe Bolboderini comprises four genera; among them, *Microtriatoma* Prosen & Martínez, 1952 has two species: *M. borbai* Lent & Wygodzinsky, 1979 and *M. trinidadensis* (Lent, 1951)^1^ ^,^ ^2^. *Microtriatoma trinidadensis* is very widely distributed and has been found in nine countries and several Brazilian states, whereas *M. borbai* has been reported only in a few states in Brazil so far^1^ ^,^ ^7^ ^,^ ^8^ ^,^ ^9^ ^,^ ^10^ ^,^ ^11^ ^,^ ^12^. 

*Microtriatoma borbai* was described based on seven male and one female specimens collected in Curitiba, Paraná state, southern Brazil. These belonged to a small population found in clusters of bromeliads, where opossums of the genus *Didelphis* Linnaeus, 1758 (Didelphimorphia, Didelphidae) and rodents were also present. One of these specimens of *M. borbai* was found to be infected with *Trypanosoma cruzi* ^1^. Rodrigues et al.^7^ found three specimens of *M. borbai* in a shelter of opossums in Campinas, São Paulo state, Brazil. Among them, one was positive for *T. cruzi* and marsupial blood. Carcavallo et al.^8^ summarized these previous occurrences of *M. borbai* and included a doubtful occurrence of the species in Goiás state, Brazil although they did not cite the source or the exact locality in this state. Alencar et al.^9^ reported the occurrence of *M. borbai* based on a male and a female specimen collected in the city and state of Rio de Janeiro, Brazil in 1963. The specimens were pinned together and deposited in the National Museum in Rio de Janeiro, without any further information about how or in which environment (e.g. a shelter) they were collected. Among 2,154 triatomine specimens collected in *Mauritia flexuosa* L.f. (Arecales, Arecaceae), a palm tree, in several locations by Gurgel-Gonçalves et al.^10^, a female specimen of *M. borbai* was found in Alto Garça, Mato Grosso state, Brazil. They hypothesized that the occurrence of *M. borbai* in *M. flexuosa* could be associated with the presence of the white-eared opossum, *Didelphis albiventris* Lund, 1840, in these palm trees.

Only eight species belonging to other three tribes of Triatominae have been reported in Espírito Santo state, Brazil so far: *Cavernicola pilosa* Barber, 1937 belonging to the tribe Cavernicolini; *Panstrongylus diasi* Pinto & Lent, 1946; *Panstrongylus geniculatus* (Latreille, 1811); *Panstrongylus megistus* (Burmeister, 1835); *Triatoma infestans* (Klug, 1834); *Triatoma tibiamaculata* (Pinto, 1926); and *Triatoma vitticeps* (Stål, 1859) belonging to the tribe Triatomini; and *Rhodnius domesticus* Neiva & Pinto, 1923 belonging to the tribe Rhodniini^11^ ^,^ ^13^ ^,^ ^14^.

When examining the private insect collection of Suzano S.A., Unidade Aracruz, the second author (DSM) asked for a loan of some specimens, one of which was identified by the first author (HRG-S) as a male specimen of *M. borbai* (Figure 1). The specimen was collected from a light trap inside a crop of *Eucalyptus urograndis* in Aracruz, Espírito Santo state, Brazil, situated at 20**°**27ʹ02,38ʹʹS 40**°**23ʹ26,17ʹʹW, by João Bosco da Silva on December 23, 1997. Considering the importance of preserving the specimen in a public scientific collection, Suzano S.A., Unidade Aracruz donated it to the Herman Lent Collection of the Coleção de Triatomíneos do Instituto Oswaldo Cruz (CTIOC), do Laboratório Nacional e Internacional de Referência em Taxonomia de Triatomíneos (LNIRTT) in Oswaldo Cruz Institute, Rio de Janeiro, Brazil; the specimen was deposited under the number 3459.

**FIGURE 1:**
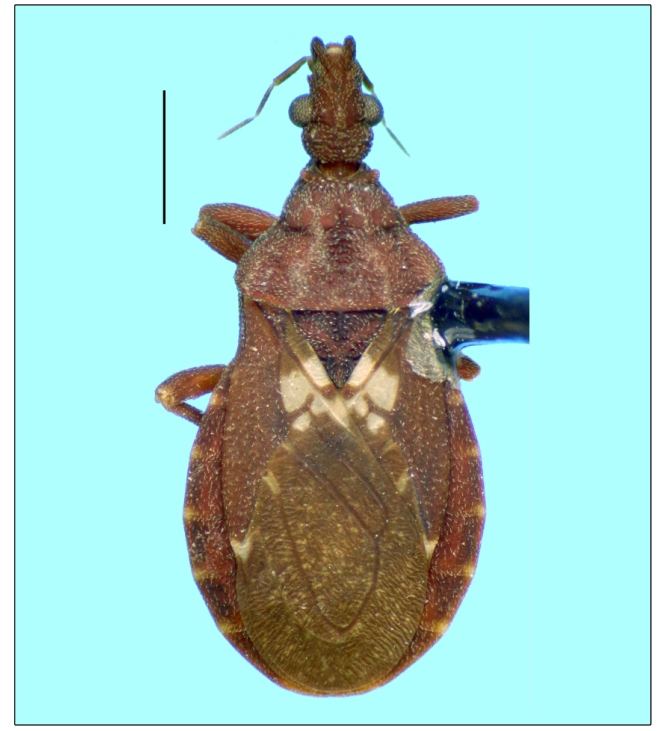
Dorsal view of the male specimen of *Microtriatoma borbai* Lent & Wygodzinsky, 1979 collected in Aracruz, Espírito Santo state, Brazil in 1997. Scale bar: 1.0 mm.

*Eucalyptus urograndis* S. T. Blake (Myrtales, Myrtaceae) is a hybrid between *Eucalyptus grandis* W. Hill ex Maiden and *Eucalyptus urophylla* S. T. Blake and is of major economic importance to the Brazilian pulp and paper industry^15^. 

The specimen of Triatominae was identified as *M. borbai* following a consultation with keys, diagnosis and descriptions available in the literature^1^ ^,^ ^13^ as well as a comparison with type specimens of *M. borbai*, which were previously deposited in the CTIOC and were examined by the first author (HRG-S). The characteristics of the specimen (Figure 1) showed complete concordance with those of *M. borbai* according to all sources of information. In short, the identification was based on the following characteristics: a small triatomine (total length of 6.5 mm) with a dorsoventrally compressed body; integument rugose covered by short setae; maxillary plates (=genae, auct.) salient, large, projecting beyond apex of clypeus; inconspicuous ocelli; short legs with thickened femora (corresponding to the tribe Bolboderini), which do not have spines (corresponding to the genus *Microtriatoma*); visible proximal and distal labial segments of the same length; and membrane of hemelytra uniformly dark, but not speckled with light and dark (corresponding to *M. borbai*). 

Notably, most previous records of *M. borbai* were based on one to three specimens only^7^ ^,^ ^9^ ^,^ ^10^, indicating that the species is somewhat rare or its populations are composed by few individuals. Therefore, although the present report of *M. borbai* in the state of Espírito Santo is based on only one specimen, it fairly supports the inclusion of this state in the geographical distribution of *M. borbai* (Figure 2).

**FIGURE 2:**
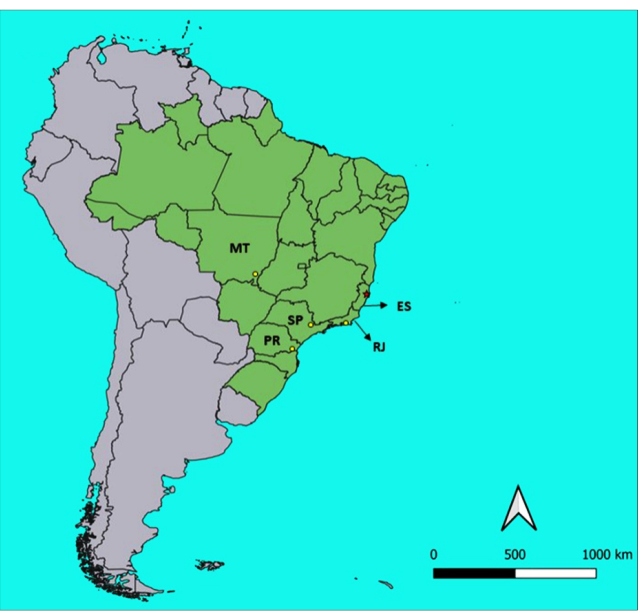
A general map of South America showing the geographical distribution of *Microtriatoma borbai*. Brazil is shown in green, and other countries are shown in gray. Locations of the previous occurrences of *M. borbai* are marked as yellow circles. The new record is indicated by the red asterisk (*). Abbreviations of Brazilian states: ES: Espírito Santo; MT: Mato Grosso; PR: Paraná; RJ: Rio de Janeiro; SP: São Paulo.

On the other hand, previous occurrences of *M. borbai* were associated with the shelters of opossums and rodents, clusters of bromeliads and *M*. *flexuosa* ^1^ ^,^ ^7^ ^,^ ^10^. Therefore, the collection of a *M. borbai* specimen inside a eucalyptus crop, which is a remarkably different environment than those reported previously, is striking. In fact, exotic tree monocultures, such as eucalyptus monocultures, are considered an important factor in reducing the composition of native species in the cultivation area^16^ ^,^ ^17^. Therefore, future studies with more intensive and diversified methods of collecting triatomine specimens should be carried out in the region to ascertain if the occurrence of *M. borbai* in a eucalyptus crop was an isolated event or if *M. borbai*, endemic to Brazil, has eventually adapted to this crop. 
